# The New ICD-11 Prolonged Grief Disorder Guidelines in Japan: Findings and Implications from Key Informant Interviews

**DOI:** 10.1007/s11013-022-09781-6

**Published:** 2022-04-27

**Authors:** Clare Killikelly, Anna Hasenöhrl, Eva-Maria Stelzer, Andreas Maercker

**Affiliations:** 1 Division Psychopathology and Clinical Intervention, Department of Psychology, University of Zürich, Binzmühlestr. 14/17, 8050 Zurich, Switzerland; 2 Department of Psychology, University of Arizona, 1503 E. University Blvd., Tucson, AZ 85721 USA

**Keywords:** Thematic analysis, ICD-11 Prolonged grief disorder, Japanese key informant interviews

## Abstract

Prolonged grief disorder (PGD) is a new mental health disorder, recently introduced in the International Classification of Diseases (ICD-11), World Health Organization Classification of Diseases (WHO). The new ICD-11 guidelines reflect an emerging wave of interest in the global applicability of mental disorders. However, the selection of diagnostic core features in different cultural contexts has yet to be determined. Currently, there is debate in the field over the global applicability of these guidelines. Using semi-structured interviews with 14 key informants, we explored the acceptability of ICD-11 guidelines for PGD according to Japanese health professionals as key informants. The interviews revealed symptoms of grief possibly missing in the ICD-11 PGD guidelines including somatization and concepts such as *hole in the heart*. Additionally, sociocultural barriers such as stigma and beliefs about the social desirability of emotions may challenge patients’ and clinicians’ acceptance of the new ICD-11 criteria.

**Supplementary Information:**

The online version contains supplementary material available at 10.1007/s11013-022-09781-6.

## Introduction

Following a proposal by an international work group, prolonged grief disorder (PGD) was introduced in the 11th edition of the International Classification of Diseases (ICD-11) in 2018 (Maercker et al. 2013). Core symptoms of PGD include persistent yearning for the deceased, preoccupation with the deceased along with accessory symptoms of emotional distress (i.e., anger, guilt, difficulty accepting the loss), impairment in daily activities, and symptoms must be present for at least 6 months after the loss (see Table 4). Historically, disordered grief has been primarily investigated in Europe and North America. Researchers from these countries are building a strong research foundation for the prevalence of PGD along with the validity and reliability of these new diagnostic criteria for the ICD-11 (Boelen et al. 2018; Maciejewski et al. 2016; Prigerson et al. 2009; Prigerson et al. 1995; Shear and Shear 2015). Nonetheless, the new ICD-11 symptom guidelines are based on the WHO’s call for increased global applicability for criteria for mental disorders (Keeley et al. 2016; Reed et al. 2011). Global applicability is established through the inclusion of cultural caveats, such as cultural norms that may prescribe the boundaries of normality and bring attention to different culturally bound symptoms. The ICD-11 definition of PGD includes consideration for cultural features and specifies that a violation of sociocultural norms regarding grief must be established before a clinical diagnosis of PGD can be made. However, in practice, the culturally relevant norms for grief may not always be clearly evident to clinicians (Stelzer et al. 2020). It remains to be determined whether the current ICD-PGD criteria for disorder reflect similar expected norms for different cultural groups.

Symptoms of mental disorder, including grief reactions, are found to differ across cultures (Kohrt et al. 2014; Rosenblatt, Walsh, and Jackson 1976). In recent years, clinicians and researchers worldwide have questioned the transferability of treatments and assessment measures between cultural groups (Canino et al. 1997; Kleinman 1978; Lewis-Fernández et al. 2017). A formal call for improved cultural validity of diagnostic systems was pioneered in 1992 when R Littlewood challenged the cultural assumptions of psychiatric diagnosis and sought clear scientific evidence to support the diagnosis and validity of these disorders in different cultures (Littlewood 1996). Littlewood asserted that the DSM is bound to the American cultural context and he emphasized the importance of considering psychiatry as a cultural system. The idea of considering ‘local psychiatrists’ is proposed by Aggarwal (2013) whereby instead of assuming that scientific findings may apply globally we must consider the cultural context in which they are developed. In response to these calls for cultural relevance, revisions of the DSM and ICD have sought to more transparently prioritize cultural validity. Indeed, the ICD-11 and the DSM –V have included cultural features and a cultural formulation interview in their diagnostic assessment tools in an effort to improve the validity of cross-cultural diagnosis (Killikelly and Maercker 2017).

A new wave of research from the fields of transcultural psychiatry, ethno psychiatry, and cultural clinical psychology is providing clear support for the importance of accurate assessment and inclusion of cultural concepts of distress (CCD) in diagnostic decision making. CCD are defined as ‘ways that cultural groups experience, understand, and communicate suffering, behavioral problems, or troubling thoughts and emotions’ (American Psychiatric Association 2013; Kohrt et al. 2014). When a questionnaire, diagnostic criteria, or intervention developed in the Global North is directly applied to a different cultural group, without consideration for the cultural groups unique expression, experience, and response to mental distress or CCDs, the validity of the assessment and acceptability of the intervention may be seriously questioned (Kirmayer and Ban 2013; Lewis-Fernandez and Kleinman 1995). There are several examples where misdiagnosis of mental disorders, treatment gaps, and reduced help seeking occurs when cultural sensitive assessments are lacking (Kirmayer, Jarvis, and Guzder 2014). Identification of CCDs can improve the validity of mental health assessment and may be particularly important in countries like Japan, where differences in grief symptoms and expression from Europe and North America are already clearly reported (Rosenblatt 2008; Shimizu et al. 2017).

An analysis of primary mental health symptoms (i.e., the prevalence of Global North-based depression symptoms) has already confirmed the presence of different language labels and types of symptoms of distress in Japan. For example, physical symptoms are important clues in the detection of mental illness in Japanese patients. Waza et al. (1999) found that 27% of the Japanese depressed patients reported only physical symptoms, whereas only 9% of the United States depressed patients reported physical symptoms. Depressed Japanese patients or patients that can be subsumed under the label “depressive disorder” were more likely to report abdominal symptoms, neck/shoulder pain, and headaches (Waza et al. 1999). When grieving, the rate of physical symptoms may be elevated in Japanese individuals (Shimizu et al. 2017). However, in the current ICD-11 criteria for PGD symptoms are mostly emotional or cognitive, and therefore may not be sufficient to capture the symptoms experienced by Japanese people. As a result, the misdiagnosis, in particular underdiagnoses might be high.

Here, we seek to explore the acceptability of the new ICD-11 PGD guidelines for professionals working with bereaved individuals. This is part of a larger research program that seeks to develop a culturally appropriate and adapted questionnaires based on the new ICD-PGD guidelines (see Killikelly et al. 2020). In the current study, we focus on the initial stages of this process: firstly, using key informant interviews and thematic analysis (see part 1 of the key informant interview in the supplementary material), we aimed explore the nature and experiences of grief including both normal and abnormal reactions from the viewpoint of professionals working with the bereaved in Japan. Secondly, we directly assessed the acceptability of the current ICD-11 guidelines and sought to identify areas where symptoms, context, or other important information are lacking (see part 2 of the interview guide in the supplementary material). The third step of the study was to develop and validate a new questionnaire including items developed from the key informant interviews. However, this questionnaire validation is beyond the remit of the current paper.

## Methods

### Research Design Overview

The key informant technique was used to obtain in-depth information on the unique experiences of professionals working in the area of grief and bereavement in Japan. The key informant technique is particularly suitable when the goal is an in-depth exploration of topics that are not well represented in the research literature, and when theory development rather than verification is of interest. This technique involves the use of key informants or community experts who provide first-hand knowledge, information, and insight on a subject within their community (Kumar 1989; Marshall 1996). This draws on the particular knowledge and understanding of community leaders and enables researchers to obtain detailed and rich data in a short period of time (Marshall 1996). Key informant interviews are often the first stage of qualitative data collection, followed by focus groups with the clinical interest group (i.e., patients with PGD). Data were analyzed using thematic analysis (see section below for more description).

### Ethics

Ethical approval was obtained the University of Zurich Faculty of Arts and Social Sciences Approval Number: 18.8.1. Ethical approval was also obtained from the Japanese host university, Jichi University.

### Researcher–Participant Relationship

Before conducting the interviews, participants were informed about the aims and nature of the study, including the experience and background of the researcher and the research team (CK, AH, AM). Informed consent was obtained. The research team was composed of students and researchers with a background in psychology (Master’s level minimum) and experience with mental health assessment. The team has extensive experience with mental health assessment. A research team in Japan was consulted during the development of the study.

### Participants and Recruitment

Japanese key informants were recruited using purposive sampling and the snowball method. To participate in the study, key informants had to be at least 20 years old, of Japanese nationality, and have professional knowledge about grief processes in Japan. We did not specifically interview bereaved individuals, but focused on the professional viewpoint first. It should be, therefore, noted that this is not a representative sample of Japanese culture, but a first exploration into the ideas of grief and bereavement according to a small sample of Japanese health care professionals. Bereaved participants will be the focus on the follow-up study. We followed the guidance from USAID on recruiting key informants in two parts: (1) Identify the local groups or organizations from which key informants should be selected (medical professionals, grief experts, and religious experts). (2) Select people from each of these categories, in addition ask individuals to suggest additional participants (Kumar 1987). We aimed to obtain a diverse sample representative of different backgrounds and viewpoints. Therefore, key informants with different roles in the community were selected, including medical staff, social workers, psychologists, grief experts, and Buddhist monks (see Table 1). The final sample comprised 14 participants (five men, nine women). For key informant interviews, 10–20 participants are recommended due to data saturation (Bolton 2008).

**Table 1:** Key informant characteristics

Profession type	Number of key informants
Palliative care doctors	3
Psychiatrist	1
Medical doctors	5
Clinical psychologists	2
Buddhist monks	2
Social worker	1

N = 14

### Data Collection

#### Study Material: Interview Guide

An interview guide was developed by the research team based on a review of the literature, the WHO “Toolkit for humanitarian settings” (World Health Organisation 2012), and in correspondence with a researcher and psychiatrist, with expertise in bereavement responses among Cambodian refugees. In the first part of the interview, normal and disordered grief responses among Japanese people were explored via 13 open-ended questions. First, participants were asked to provide a detailed description of the grief reactions typically experienced by Japanese adults following the death of a loved one. *‘What are the grief reactions of someone who has experienced the loss of someone close in Japan? Please give me as many answers as you can think of.’ ‘The severity of grief responses after losing someone close varies over people. When you think about your family, friends, or patients, from which degree of severity of grief response would you worry about the person who is grieving?’’* In the second part of the interview, participants were asked to specifically reflect on the grief-related difficulties bereaved Japanese individuals face and how this would be impacted by the new PGD guidelines. *‘What is the merit or demerit of introducing a new disorder to Japanese culture?’ ‘What might be the cause of the occurrence of an abnormal response to grief? (Type of loss, type of person: Gender, Beliefs, Spirituality, religion).’* With regard to disordered grief, participants were asked to discuss the duration and degree of impairment, as well as differential diagnosis (‘*How is disordered grief different from depression and other mental health disorders?*’). Further questions pertained to the perceived causes of disordered grief, and the coping strategies endorsed by Japanese bereaved people, such as remembering the person who had died, or social support.

### Procedure: Key Informant Interviews

Prior to the in-person interview, the researcher (AH) emailed the interview guide to the key informant. During the key informant interview, participants first received relevant study-relevant information and completed a consent form. This introduction was followed by the semi-structured interview described previously (see Supplementary Material). All interviews were conducted in Japanese and in person at the place of choice of the interviewee, i.e., at home, local café, or at the university.

### Data Analysis

Interviews were transcribed verbatim (in Japanese) and then analyzed in Japanese by two psychologists fluent in both English and Japanese using the qualitative software program (AH, KS), MAXQDA 2018 VERBI, 2017. Thematic analysis, a qualitative data analysis method for identifying, analyzing, and reporting patterns across a data set, was used (Braun and Clarke 2006). Thematic analysis follows clearly prescribed steps or phases (Braun and Clarke 2006) involving familiarization with the data, generating the initial codes and themes, reviewing, defining and names themes, as well as writing up the findings. Reading the transcripts in detail helped to familiarize the researchers with the data and to generate the initial codes. Based on the perceived relationship between codes, they were then sorted into potential themes and reviewed for coherence within and distinctiveness between themes. Themes were defined as data that contribute to answering our research questions. Themes were identified both inductively and deductively. For example, the themes of *social affects, close relationships, and culture and society* represent deductive themes extracted from the literature (Maercker and Hecker 2016). These themes organized various subthemes, which were derived inductively (e.g., emotional reactions, cognitions, social emotions) based on the interview texts. In addition, we employed a latent content-analytic approach, which identifies or examines underlying ideas regarding what has been said (Braun and Clarke 2006). Such a latent approach embeds the grief reactions in the sociocultural context. Lastly, the assumed core essence of each theme was extracted. For each individual theme, a detailed definition was created, the theme was named, and we considered how each theme fit into the context of the data and how it related to the research question.

### Methodological Integrity

The interviewer guide was initially drafted in English and reviewed by an expert in the field to ensure fit with regard to research aims (KS). The final version of the English interview guide was then translated in Japanese by two independent professional translators. Together with a Japanese researcher (KS), the two Japanese translations were compared and the most fitting version was selected. The interview guide was then piloted with two Japanese participants to test its appropriateness and identify potential limitations. Based on the feedback from the pilot test, we modified the interview guide. For example, because there is no commonly used word for grief in the Japanese language, we decided to provide a thorough introduction to the study topic at the beginning of each interview. In addition, we deleted the question, “Are there any aspects of the background or identity of bereaved people that might impact individuals’ grief responses?” Questions about background or identity are likely difficult to answer, as many participants might have never considered the meaning of their own identity or background. To ensure the quality of the results of this study, the guidelines for qualitative research studies developed by Elliott et al. (1999) were applied including the use of multiple qualitative analysts for verification and coherence credibility. In line with the investigator triangulation method (i.e., data are analyzed by multiple researchers to balance the interpretative bias of an individual researcher) (Patton 1999), two researchers independently coded the data to review the data for discrepancies, overstatements, or error. The analyzed interviews were then compared and obscurities and deviations were discussed until consensus was achieved. Furthermore, coherence was achieved by integrating the identified grief reactions into the sociocultural environment of Japan and by developing a model that fits with the underlying structure of the domain of grief in Japan. Data saturation in this context is defined as the identification and reduction of thematic codes based on the concept of information redundancy (see Braun and Clarke 2019 for more information) (Braun and Clarke 2019).

## Results

These results are drawn from responses to both part 1 and 2 of the interview guide. Because of the extensive findings, we have streamlined the results according to the socio-interpersonal view of PTSD (Maercker and Hecker 2016). Therefore, although the codes emerged across the whole data set in an inductive manner, we have structured the results deductively using the socio-interpersonal framework. Here, we present firstly, the results related to the individuals experiences of grief, i.e., the similarities and differences between the new ICD-11 grief symptoms and descriptions of grief from Japanese key informants. Secondly, we explored wider cultural–societal themes-related social affects, beliefs, and values that may impact on the experience, expression, and acceptability of PGD.

### Grief Symptoms in the Individual

This part of the analysis focused on establishing common grief symptoms for disordered grief in Japanese bereaved. Participants described a range of *emotional responses* that are associated with both normal and abnormal grief responses. In terms of specific emotional reactions, the following grief reactions were identified; longing-yearning, sadness, depression, feelings of solitude/sense of loss, pain, manic defense, numbness, exhaustion, crying, and apathy. Longing/yearning for the deceased was the most frequent emotional reaction reported and was referred to as missing the deceased, yearning to make interpersonal connections with the deceased, feeling lonely because of the absence of the deceased, and wanting to be reunited with the deceased. Sadness was the second most frequent grief reaction reported by the participants. Some of the participants noted that sadness could be intensified by risk factors, such as the nature of the relationship with the deceased, gender, or a lack of social support. The sense of loss was described using a metaphor for distress ‘as a hole opening up inside the *kokoro* (heart).’

Participants also identified several different *cognitions or thought patterns* that were associated with grief. These codes included common and helpful cognitions as well as potentially pathological cognitive responses. In terms of positive or helpful cognitions, participants noted thoughts of acceptance and personal growth after experiencing someone’s death can occur. Participants described the acceptance of the death as something that must be achieved in order to cope with the death. Overcoming this hurdle would take time; however, once it was overcome, the bereaved would be able to move forward. If the bereaved were not able to accept the death, this lack of acceptance could operate as a risk factor and trigger PGD. Cognitions could also be future-oriented (inability to find happiness, fear that the person who died is being forgotten, loss of meaning or purpose in life) or associated with negative feelings such as feeling not understood, feeling of being punished, feeling worthless, or preoccupation/worry and in some cases the experience of hallucinations and delusions. Additionally, the feeling of being punished and the fear that the person who died would be forgotten were mentioned in connection with losing a child.

Participants identified the following *bodily reactions*, starting with those most frequently mentioned: sleep disturbances, loss of appetite, eating disorders, stomach ache, headache, nausea, diarrhea, weight loss, and palpitations. By far, the most frequently mentioned somatic reactions were sleep disturbances (16 codings across 10 participants) and loss of appetite (16 codings across 10 participants), followed by stomachache (eight codings across two participants) and headache (six codings across three participants). Feeling sluggish was associated with increased impairment of functioning, such as no longer bathing or cleaning the house. One key informant said that it would be easier for Japanese bereaved to express physical symptoms than emotions: *Maybe it is easy to say it. It is difficult to express emotional feelings, even for me. So, I think it is easier to express physical [symptoms]. The body, for example, physical symptoms, is easy to understand.—Participant 6.* See Table 2 for an overview of the symptoms described by participants.

**Table 2:** Common symptoms of grief identified at the individual level

Grief symptoms in the individual			
Subthemes	Emotional responses	Cognitive responses	**Somatic responses**
Codings	Longing/Yearning Sadness Depression Pain Sense of loss **Manic defence** Numbness **Exhaustion** Crying **Apathy** **a hole opening up inside the *kokoro***	**Acceptance** **Personal growth** Inability to find happiness **Fear that the person who died is being forgotten** Loss of meaning or purpose in life **Feeling not understood** **Feeling of being punished** Feeling worthless Feelings of solitude Worry Preoccupation	Sleep disturbance Loss of appetite Eating disorders Stomach ache Headache Nausea diarrhea Weight loss Palpitations Sluggishness, dullness

^*^Bold indicates differences from ICD-11

### Exploration of Cultural/Societal Factors

This part of the analysis is focused on exploring the beliefs and values and other cultural factors, identified by key informants, as common in Japanese people particularly in the context of grief. Thematic analysis of these questions revealed three levels of sociocultural influence specific to the Japanese culture; *Social affects* (content related to subthemes emotional control and social emotions), *Close Relationships* (subthemes: upbringing, gender, social support, and continuing bonds), and *Culture and Society* (subthemes: social isolation, changing role of religion, and stigma). The relationship between these emerging levels is illustrated in Figure S1 (in supplementary material).

### Social Affect

In this study, *social affect* comprises two subthemes: emotional control and social emotions. The subtheme of emotional control, beginning with the most frequently occurring codes, comprises suppression of emotional expression, self-reliance, avoidance of reminders of the deceased, and denial. In terms of the subtheme, suppression of emotional expression is often related to actively withholding emotional expression when in public or in social situations. For example, it was frequently mentioned that bereaved people do not want to show weakness or do not want to bother the people around them. Additionally, participants reported that some bereaved stopped contacting friends and acquaintances during times of suffering. This is related to the subtheme of ‘self-reliance.’ It was reported by participants that Japanese people do not want to accept support from outside—they would rather prefer to overcome their grief by themselves. Another topic that arose was that death is a taboo in Japan and people therefore avoid talking about death most of the time. Denial was reported as bereaved ‘people fleeing from the memory of the deceased or not wanting to recognize their own feelings.’ Interestingly, there was a consensus among some participants that suppression of emotional expression, self-reliance, and denial were unhealthy responses. Some participants identified that avoidance of the expression of feelings to others could cause a worsening or prolonging of the symptoms, while talking about one’s distress would help with acceptance of the death: *“A lot of people I met wanted to kill their feelings, but couldn't, so they became strange afterwards.... After a long time has passed, they tell me: “In reality, it was like this: I killed my own feelings. They [the bereaved] lived without letting their feelings out.”—Participant 7 [The bereaved] can gradually accept the death by becoming close to other people and talking about their suffering.—*Participant 1.

Participants stated that not expressing one’s own feelings could be a cause of regret as the bereaved had not expressed their feelings towards the deceased while the deceased was still alive. *Japanese people do not touch each other, give hugs, and almost never greet. So normally, Japanese people do not say, “I love you” or “I care about you.” So they feel regret.—*Participant 11. This is also related to the second subtheme, social emotions. The most frequently occurring codes, in order of occurrence, were anger, blame, regret, self-condemnation, and shame. Anger towards others and the event and blaming others were described as interrelated and possible indicators of disordered grief. However, to blame oneself rather than others was coded as self-condemnation. Self-condemnation was mentioned as especially prevalent in mothers who lost a child, for example, due to a miscarriage. Regret was portrayed as not having loved the deceased enough while he or she was still alive, or a regret for having done something bad or not giving or doing enough to the deceased while he or she was alive. Regret was presented as being very prevalent during the grieving process in Japan and that it was propagated through the Japanese culture of not expressing one’s feelings. Another term for regret in Japanese is *kokoro nokori*, which literally means heart residue or remains of the heart. Participants reported that Japan is considered to have a culture of shame, and that shame would occur when expressing one’s feelings, such as sadness, to others as identified above under the theme of emotional control.

### Close Relationships

This interpersonal level includes influencing factors from the individuals’ immediate relationships that may shape their grief. This includes all themes considered by the individual as in-group (i.e., family, close others, meaning inside their close social network). These subthemes included upbringing, gender, social support, and continuing bonds. Differences in upbringing were reported as a possible cause of PGD (Table 3).

**Table 3:** Exemplar quotes from participants representing each subtheme

Themes	Exemplar quotes
*Close relationships*	
Upbringing	*There is a connection [to disordered grief] with the family functioning of the bereaved. Whether the [relationship with the] father and mother functioned properly or not. Whether the family functioning in the early childhood was favorable or not....Other than this, the personality also [has a connection to disordered grief].—Participant 5*
Gender	*Japanese men leave everything up to women: cooking, washing, how to care for money, or how to get cash at the bank. So, after the wife dies, it is very difficult for the husband. All my female cancer patients return home at least once before they die. They say: “If I die, my husband will have troubles. I have to prepare him.”— Participant 6*
Social support	*If there is a person who can be asked for advice, the kokoro [heart, mind] is supported. A person who listens carefully is the most important.—Participant 1* *When my friend lost her mother, she didn’t want to contact me for about a year. Maybe she wanted to digest the sadness on her own.—All by herself, isn't it painful?—Hmm, well she’s married and has a child, so they are always together. She could find resolution in this. She can talk to uchi [inside]but didn't want to contact soto [outside].... Of course, I was worried but she didn’t want me to enter. She has been my friend since primary school but she didn’t want me to enter.... For example, if somebody loses his/her mother but the other person still has both of his/her parents, the standpoint is different, we cannot come together, the feelings cannot get close to each other.—Participant 10* *If there were a place for discussion between families or people who experienced something similar and have the same standpoint, it would be wonderful.—Participant 12*
Continuing bonds	*For us Japanese people, when we are asked about the difference between a grave and butsudan, it is the way we use them. The grave is like a symbol, the butsudan is not a symbol. [In front of the butsudan, the bereaved] can address [the deceased], look at pictures and give food. The deceased is at one’s side.... So, also for Japanese people, the opportunity to talk is important. Please talk! Even if there is no grave or butsudan.—Participant 4*
*Culture and society*	
Social isolation and modernization	*Do you know the word dokkyorōjin? It means elderly people living alone without family. They are planning their own deaths, their graves, and so on. There are companies that make preparations for their death but many people are dying alone. Some people are dying in their homes, but many are dying in institutions. They are going to institutions to die because there is no caretaker, no family. I think it’s quite sad.— Participant 6* *The changing of the family structure is an important point. Up to now, the extended family mainly lived together; grandmother, grandfather, parents, and children. But nowadays, it’s the nuclear family, one or two children, and the parents are very old. Or there are only two people living together or the family is scattered around different places and people are living alone.—Participant 9* *In Japan, there is the Buddhist memorial service, the first anniversary of a person’s death, the second anniversary of a person’s death, and so on. People meet at the anniversary service and remember the person who died. Fellow people who gather to remember the person. By talking about how this person was, [the bereaved] doesn’t feel all alone, there are other people who think about the person, too and the kokoro[heart, mind] can settle down.—Participant 8* *Yet, it may be difficult to participate in these rituals, such as attending funerals, death* *anniversaries, or visiting graves as many people might have left their hometown to live in the city. The grave is located at one’s birthplace. So, when people move to Tokyo to work, it is difficult to visit the grave.—Participant 2*
The changing role of religion	*There is one problem with religion: of course, there are people who properly believe, but most of the people say: “My uchi [here: family] is more or less Buddhist.” One* *only really becomes aware of religion at the time of the funeral or when visiting the* *grave. In Japan, there are events at the change of seasons like o bon, but if you’re not* *participating in these events, you don’t have an awareness of being Buddhist. So,* *compared to Europe and North America, religion cannot support the overcoming of* *grief. When a Buddhist monk chants at a funeral, people don’t understand the words* *he is saying. So, when you don’t understand the words at all, [the ritual] is reduced to* *a formality.—Participant 3* *The funeral is helpful. It is a process that allows you to put your own feelings in order* *Putting them in order and preparing for the farewell. Being busy [during the time* *of the funeral] is also a good thing, well; I think there is at least one good thing:* *There is no free time to feel sad when [you are] busy.—Participant 6* *In Japan, there is the Buddhist memorial service to remember the deceased, like, for* *example, the first death anniversary or the third death anniversary. Remembering the* *deceased, gathering together with fellow bereaved people, to talk about the deceased,* *[to remember] that there is not only you, there are other people who remember the* *deceased as well. It calms down the heart.—Participant 12*
Stigma	*It would become a considerable economic burden when somebody in the family commits suicide by jumping in front of a train, as the railroad company would demand that the family pay compensation for the damage caused by the suicide. In Japan, when somebody jumps in front of a train to commit suicide, the railroad company demand damage compensation. They claim it from the bereaved family. The family will suffer financially and they will suffer from losing the person who committed suicide.—Participant 12* *When I have to refer patients to the Department of Psychiatry, they have to overcome* *a hurdle. When I introduce them to the Department of Psychiatry, their facial expression* *darkens. It is difficult.—Participant 9* *[Japanese people] would absolutely never say to other people: “In my family, someone* *committed suicide.” There is the stigma regarding suicide, even for Japanese* *people. There were a lot of people dying for the Emperor of Japan, committing suicide* *In the earlier days, suicide was considered something beautiful. Maybe this* *changed in the period after the Second World War?—Participant 4* *Japanese people avoid talking about death. If there were an increase in the number of* *organizations that did this kind of care, it would be nice, but for now, these organizations* *are rare, so not everybody can receive [the care]. It is a problem in society.—* *Participant 7* *People do not go to the hospital when they experience severe grief responses. There is* *strong resistance. People do not want to go to the hospital.—Participant 2*

Important gender differences were also noted. For example, several participants noted that men have more trouble expressing their feelings in front of others or would feel ashamed showing them and therefore do not attend therapy sessions or self-help groups. Men are more likely to avoid reminders of the deceased or try to escape from their feelings. Coping with the loss of a partner is more difficult for men (Table 3). For women, coping with the loss of an unborn child is more difficult than for men. The key informants also stated that when they lose a partner, women have economic troubles because in Japan it is mostly men who practice a profession. In order to cope with the loss of a loved one, social support was reported as being the most important factor; a lack of social support would increase the risk of PGD. The key informants recognized family sessions or group sessions as being the most welcomed therapy for Japanese bereaved people to overcome grief (Table 3). One of the most prevalent themes in the interviews was maintaining bonds with the deceased, which are supported by rituals, such as talking to the deceased everyday at the butsudan (Buddhist household altar), or remembering the deceased by visiting the grave or celebrating the death anniversary. For Japanese people, traditionally, because there is the grave and the butsudan, it is easy to talk to the deceased (Table 3).

### Culture and Society

The final category is the level of culture and larger society. This relates to factors present in the larger sociocultural sphere that may impact grief expression and understanding. Subthemes that emerged within this category included social isolation due to urbanization and migration, as well as the changing role of religion and stigma. Several participants noted that factors associated with modernization such as increased urbanization and migration have led to increased social isolation. One participant noted that while in rural areas in Japan, it would be common for neighbors to support the bereaved family, for example, by helping to organize the funeral, in urban areas, it is often necessary to hire a company to help organize the funeral because of the lack of support from the family or community (Table 3). Migration and the spread of the nuclear family has also led to increased isolation whereby the traditional death rituals cannot be performed as intended (Table 3). In Japan, most people would not consider themselves religious. Nonetheless, rituals such as Buddhist funerals or a visit to a temple or a shrine at New Year remain the norm. This conflict may lead to the feeling that the traditional death rituals feel like empty formalities as one of the key informants stated, see exemplar quote participant 3. When the key informants were asked if they thought that a funeral would be helpful to Japanese people or would be a burden, some of them answered that it would be helpful and that organizing the funeral would keep the bereaved family distracted and protect them from feeling too sad (see quote of participant 6, Table 3). The key informants reported that visiting the grave, the funeral, the annual death anniversary, and praying at the butsudan could help to maintain social support for other bereaved people (see quote of participant 12, Table 3).

Stigmatization was discussed as a barrier to adjust to loss and was reported for prolonged grief, mental diseases, and death through suicide. Even though bereavement services are offered by hospitals or psychiatry departments key informants reported that, in Japan, people avoid going to the hospital or other mental health care providers when experiencing disordered grief due to stigmatization of mental diseases (see quote of participant 2, Table 3).

Finally, based on the themes and subthemes that emerged across the whole data set, Table 4 presents a summary of the recommendations from the key informants and how the ICD-11 PGD guidelines might be adapted for the Japanese context.

**Table 4:** Guidelines for prolonged grief disorder proposed in the ICD-11 and adaptations of PGD in Japanese bereaved adults

PGD as proposed in the ICD-11 (Killikelly and Maercker 2017)		Adaptation to PGD for Japanese bereaved adults	
A. At least one of the following:	1. Persistent and pervasive longing for the deceased or 2. A persistent and pervasive preoccupation with the deceased	A Core items	A persistent and pervasive preoccupation with the deceased
B. Examples of intense emotional pain	Accompanied by intense emotional pain, e.g., sadness, guilt, anger, denial, blame, difficulty accepting the death, feeling one has lost a part of one’s self, an inability to experience positive mood, emotional numbness, difficulty in engaging with social or other activities	B. Examples of somatic reactions, intense emotional pain, and harmful behavior	Accompanied by somatic reactions, e.g., disturbed sleep, disturbed eating, headaches, stomach ache, feeling sluggish Accompanied by intense emotional pain, e.g., sadness, regret, anger, blame, difficulty accepting the death, feeling one has lost a part of one’s self, emotional numbness, difficulty in engaging in social or other activities, avoidance of expression of feelings towards others, feeling not understood, hallucinations, or illusions Accompanied by harmful behavior, e.g., secluding oneself at home, not bathing or cleaning, not contacting family or friends, losing control of one’s feelings, neglect of one’s appearance, suicide attempts, self-injury
C. Time and impairment criteria	Persisted for an abnormally long period of time (more than 6 months at a minimum) following the loss, clearly exceeding expected social, cultural, or religious norms for the individual’s culture and context. Grief reactions that have persisted for longer periods that are within a normative period of grieving given the person’s cultural and religious context are viewed as normal bereavement responses and are not assigned a diagnosis The disturbance causes significant impairment in personal, family, social, educational, occupational, or other important areas of functioning	C. Time and impairment criteria	The disturbance causes significant impairment in personal, family, social, educational, occupational, or other important areas of functioning. Impairment of functioning includes an inability to lead a full or normal life, no longer going to work or school, not getting out of bed in the morning, neglecting appearance, and the necessity for medication

## Discussion

This research used the key informant approach to better understand symptoms of grief in Japan from the perspective of local health care professionals. This study revealed important findings that may significantly impact on the acceptability and implementation of the new ICD-11 guidelines for PGD.

For many years there has been a reliance on assessment measures developed in North America and Europe. A review by Hollifield et al. (2002) found that 78% of studies relied on measures developed in the Global North to examine individuals’ mental health. One cannot assume that an assessment measure developed for North American and European populations will be valid and accurate in a different cultural context. The current study confirms that some of the symptoms of grief in Japan are different from the core symptoms outlined in the ICD-11 in notable ways. Somatic symptoms were found to be prominent. The importance of considering somatic symptoms is supported by additional research on depression in Asia (Gureje et al. 1997; Okazaki 2000). In a study comparing symptoms in those diagnosed with depression, 27% of the Japanese patients reported physical symptoms only, while 9% of the U.S. patients reported physical symptoms only. Depressed Japanese patients were more likely to specifically report abdominal symptoms, neck/shoulder pain, and headaches (Waza et al. 1999). Interestingly, a study by Zhou et al. (2016) explored somatic symptoms in Chinese and North American patients with depression. They concluded that both cultural groups experience somatization. However, this may have two different origins: from the lived experience of physical sensations/physiological response, or it may also be a strategy to access support and avoid stigma. Individuals with depression experience a range of symptoms, but perhaps choose to emphasize the somatic ones when speaking with others or seeking help from others because somatic symptoms are understood to be less socially problematic than psychological symptoms (Zhou et al. 2016). Because of the greater presentation of somatic symptoms, diagnostic findings on the phenomenology of mental illnesses in Japan might be quite different from non-Japanese samples. The symptoms included in Western instruments may not be sufficient to capture the somatic distresses experienced by people in East Asian samples (Waza et al. 1999). This is important to consider in terms of the ICD-11 definition of PGD but also for the DSM-V’s persistent complex bereavement diagnosis. Currently, this diagnosis is under review but the inclusion of cultural features would improve the global applicability of this definition. A team of the Netherlands are developing the Grief and Bereavement Cultural interview that may supplement the DSM-V cultural formulation interview (Smid et al. 2018).

Another predominant grief reaction identified by key informants was longing and yearning for the deceased. Although this is a core item in the ICD-11 definition of PGD, this is seen as a normal response in the Japanese context. In Japan, this is associated with culturally specific healthy behaviors such as performance of rituals after the death and the maintenance of continuous bonds. The importance of maintaining continuous bonds with the deceased in Japan is described by Klass (2001): ancestor worship helps the bereaved to realize that the deceased is dead and to resolve any regret within the relationship. The longing and yearning reported by the key informants could be thus an important part of the normal bereavement process and should not be considered a problematic response.

Emerging themes also revealed the importance of considering the broader social–cultural landscape and its impact on the expression of mental disorder. The socio-interpersonal model of PTSD attempts to capture the importance of social context on the experience and expression of stress-related disorders, including PTSD (Maercker and Hecker 2016) and adjustment disorder (Lorenz et al. 2018), and could also be applied to PGD. The current findings are mapped onto this model—in a modified and extended version—in Fig. 1 supplementary material.

In this study, social affects comprise the two subthemes: emotional control and social emotions. Mauss et al. (2010) describe emotional control as a self-regulatory process, which regulates emotional responses, for example during a social interaction. Previous research suggests that Asian culture values emotion control (i.e., modulating one’s own emotional experiences and expressions) more than European-American culture (Tsai et al. 2006). Several key informants reported that Japanese people do not want to show or talk about their feelings. This finding is supported by Kim (2015) who found that in Japanese culture, it is deemed inconsiderate to openly share private feelings. Grief and sadness are usually not expressed to others and should be dealt with independently. Showing emotions such as sadness at a funeral, for example, would discomfort the guests and might be considered to be impolite (Kim 2015). This difference in emotional control strategies may impact on the willingness of Japanese bereaved to report symptoms of grief. Several researchers have noted ‘hidden grief,’ i.e., lack of reporting of grief distress, particularly after a death by suicide (Tsukahara et al. 2016).

Following the death of a loved one, bereaved individuals experience a range of intense emotions. Social emotions are emotions that necessarily depend on other people’s thoughts, feelings, or actions, and they are often linked to social behaviors (Hareli and Parkinson, 2008). In this study, social emotions were reported including anger, blame, regret, self-condemnation, and shame. Markus and Kitayama (1991) stated that self-serving emotions such as anger are usually derived from an independent view of the self. As Japan has historically been described as a collectivist culture with an interdependent sense of identity, self-serving emotions may be less common. Instead, other-serving emotions such as guilt or shame may be expressed (Markus and Kitayama 1991). The social emotions common in Japan may include regret, self-condemnation, and shame (Kitayama et al. 2006). Accordingly, key informants in this study reported that Japanese bereaved adults would not be expected to express anger or blame. However, in the case that it is expressed, this may violate a cultural norm and be a possible indicator of disordered grief.

*Close relationships and culture and society* relate to wider themes regarding Japanese social influences and the social context. Key informants in the current study made a clear distinction from where support might be sought; preferring the inside network of close relationships as opposed to the outside external network such as health care professionals. Help-seeking in Japan resides at this social, inside level. In the study of Asai et al. (2012) social sharing, including talking about feelings and seeking emotional support from close friends and family, was identified as a helpful coping strategy. Therefore, when treating PGD in Japan, the inclusion of social support and family in therapy should be considered.

The level of culture and society also revealed the importance of considering social changes in society. While traditional values and rituals are still recognized in Japan, social change, including the dispersion of the nuclear family, might disrupt the bereavement process for those who expect to mourn in the traditional sense with family and relative members (Shimizu et al. 2017).

This exploration of grief reveals the deep complexity of the issue of cultural acceptability of the new ICD-11 PGD guidelines. There are several layers that may influence the expression and experience of grief and clinicians should consider that a simple listing of cultural features may not capture the true depth of grief. The qualitative analysis provided insight for clinical application, for example, due to prominent values of emotional control, stigma towards mental illness, or lack of somatic items in the assessment measure, PGD may be underestimated in Japanese culture with the current ICD-11 PGD guidelines.

### Limitations

There are several important limitations in the current study. One important limitation may be that the interview guide prompted key informants to identify differences between the ICD-11 symptoms and their understanding of grief, in terms of boarder Japanese culture. This may have biased their responses to yield more general, perhaps stereotypical, comments instead of comments grounded in their own individual experiences. Additionally, the key informant sample includes several medical professionals and a limited number of grief experts outside of the medical sphere. We only included one religious expert, a Buddhist monk. Different religious leaders may have different viewpoints on bereavement norms and types of support. In a follow-up study further interviews or focus groups should be conducted with different religious leaders, grief counselors as well as bereaved individuals.

### Implications and Conclusions

This study is the first to explore the new ICD-11 PGD guidelines from the perspective of Japanese bereavement professionals. Through the use of multiple levels of qualitative analysis, this study aims bridge research and practice by providing insight into the symptoms of grief in Japan along with better understanding of wider cross-cultural differences. For example, somatic symptoms are robustly endorsed in the Japanese context, however, these are largely missing from the PGD ICD-11 definition. On the other hand, yearning and longing for the deceased (a core symptom of PGD ICD-11) is considered a normal and encouraged process, related to the emphasis on continuous bonds. Clinicians will need to consider these possible cultural differences before diagnosing PGD in the Japanese context. Considering the deeper beliefs and values of a culture and how this may impact on the assessment of grief is of great importance*.* For example, the current study explored PGD in terms of the socio-interpersonal model of stress and highlighted how cultural factors such as stigma and the high regard control of emotions may provide clinicians with challenges when assessing PGD. In conclusion, this is the first study to explore the voice of bereavement professionals in Japan as the new ICD-11 PGD definition is introduced globally. We would encourage clinicians to follow the current ICD-PGD definition in order to provide a formal diagnosis, however, when treatment planning and allocating support for the individuals the wider cultural context must be considered (Killikelly et al. 2020).

## Supplementary Information

Below is the link to the electronic supplementary material.

Supplementary file1 (DOCX 43 kb)
