# Supplementary material for: The New ICD-11 Prolonged Grief Disorder Guidelines in Japan: Findings and Implications from Key Informant Interviews
Source: Cult Med Psychiatry. 2022 Apr 27;47(2):519–42. doi: 10.1007/s11013-022-09781-6 (PMC10167141; doi:10.1007/s11013-022-09781-6)
Supplement: Supplementary file 1 — Supplementary file1 (DOCX 43 kb) [file 11013_2022_9781_MOESM1_ESM.docx]

# Supplmentary Material: Key Informant Interview (English version)

1. **Key Informant Interview - First Part**

The loss of someone close is a common but difficult life experience. Most people are able to adapt, while others struggle with long-term intense distress and suffering.

The severe and long-lasting impairment caused by the death or loss of a beloved one is described as Prolonged Grief Disorder (PGD) in the new Classification of Diseases (ICD-11) from the World Health Organization (WHO). We want to examine, whether the criteria for this new disorder is applicable for Japanese people or not. The goal is to develop an instrument to measure prolonged grief disorder. In this interview, I would like to know about the experience of Japanese people in general after experiencing the death of someone close. I am interested in your professional opinion about bereaved Japanese people, and NOT your own personal experience of losing someone close. There is no right or wrong answer.

**Open Questions**

1. What are the difficulties of Japanese who have experienced a death of a beloved one? Please give me as many answers as you can think of.
2. What are the grief reactions of someone who has experienced the loss of someone close in Japan? Please give me as many answers as you can think of.

**Disordered Grief**

1. The severity of grief responses after losing someone close varies over people. When you think about your family, friends, or patients, from which degree of severity of grief response would you worry about the person who is grieving?
    What is disordered duration?

What is the degree of impairment (across which domains of functioning, all or only culturally relevant areas)?

1. In your opinion, what should be done when a disordered grief response occurs?
2. How would a disorder of grief be different from depression or other mental health disorder?
3. What is the merit or demerit of introducing a new disorder to Japanese culture?

**Cause**

1. What might be the cause of the occurrence of an abnormal response to grief? (Type of loss, type of person? Gender, Sexual Orientation, Believes, Spirituality, religion)

**Coping**

1. How do people remember the person who died?
2. Do you think that this remembrance is helpful to Japanese people or becomes a burden?
3. I want to ask you about support for bereaved people in Japan. In your opinion, what kind of support do bereaved people wish for in Japan?
4. Is there any support for the overcoming of difficulties after experiencing a death of a beloved?
5. Does anything prevent Japanese bereaved adults from getting the help they need?
   (For example, money, work or family commitments, stigma or discrimination, or lack of services?)
6. **Assessment of the PGD Criteria Proposed for the ICD-11**

The severe and long-lasting impairment caused by the death or loss of a beloved one is described as Prolonged Grief Disorder (PGD) in the new Classification of Diseases (ICD-11) from the World Health Organization (WHO). In the following, we would like to know if the proposed criterions are applicable for assessing PGD in Japan. You will be asked if criterions should either be included, removed or changed. If you feel a criterion should be removed or changed, please specify why or how.

Below is an overview of the criterions. In the following, you will be asked about every criterion separately.

1. Should the criterion be included?
2. Should the criterion be removed?
3. Should the criterion be changed? How?

| Prolonged grief disorder proposed for the ICD-11 | |
| --- | --- |
| Event | Person experienced the death of someone close at least six months previously |
| A. At least one of the following: | 1. Persistent and pervasive longing for the deceased  or  2. A persistent and pervasive preoccupation with the deceased |
| B. Examples of intense emotional pain | Accompanied by intense emotional pain e.g. sadness, guilt, anger, denial, blame , difficulty accepting the death  Feeling one has lost a part of one’s self An inability to experience positive mood  Emotional numbness  Difficulty in engaging with social or other activities |
| C. Time and impairment criterion | Persisted for an abnormally long period of time (more than 6 months at a minimum) following the loss, clearly exceeding expected social, cultural or religious norms for the individual's culture and context. Grief reactions that have persisted for longer periods that are within a normative period of grieving given the person's cultural and religious context are viewed as normal bereavement responses and are not assigned a diagnosis.  The disturbance causes significant impairment in personal, family, social, educational, occupational or other important areas of functioning. |
| ICD-11: International Classification of Diseases (11th edition). | |

1. **Questionnaire Development**

In the following you can find an example of an instrument to measure Prolonged Grief. I would like to know your opinion on this scale, if this would be used in Japan.

What needs to be asked in a questionnaire measuring grief in Japan?

- Direct / indirect questions?
- Short / long questions?
- Few / many questions?
- Could we use a computerized questionnaire?
- How should the choices of answers be formulated?
- How would a measure for Japanese be different to one for a European/American population?

**International Prolonged Grief Scale -** Killkelly and Maercker (2018), integration the PG-13 (Prigerson, Vanderwerker and Maciejewski, 2008) and the SCI-CG (Bui et al., 2015)

Instructions**:** Please mark the box next to the answer that best describes how you have been feeling over the past month.

|  | Almost never  (less than once a month)  (1) | Rarely  (monthly)  (2) | Sometimes (weekly)  (3) | Often (daily)  (4) | Always (several times a day)  (5) |
| --- | --- | --- | --- | --- | --- |
| 1. In the past month, how often have you felt yourself longing or yearning for the person you lost? | 1 | 2 | 3 | 4 | 5 |
| 1. How often have you felt yourself lost or absorbed in thoughts or daydreams about the deceased person? | 1 | 2 | 3 | 4 | 5 |
| 1. In the past month, how often have you had intense feelings of emotional pain, or sorrow, related to the lost relationship? | 1 | 2 | 3 | 4 | 5 |
| 1. Have you had intense feelings of guilt about the death or circumstances surrounding the death? | 1 | 2 | 3 | 4 | 5 |
| 1. Do you feel bitter over your loss? | 1 | 2 | 3 | 4 | 5 |
| 1. In the past month, how often have you tried to avoid reminders that the person you lost is gone? | 1 | 2 | 3 | 4 | 5 |
| 1. Do you find yourself blaming others or the circumstances for the death? | 1 | 2 | 3 | 4 | 5 |
| 1. Have you had trouble accepting the loss? | 1 | 2 | 3 | 4 | 5 |
| 1. Do you feel that you have lost a part of yourself? | 1 | 2 | 3 | 4 | 5 |
| 1. It is very hard to experience joy or satisfaction | 1 | 2 | 3 | 4 | 5 |
| 1. In the past month, how often have you felt stunned, shocked, or dazed by your loss? | 1 | 2 | 3 | 4 | 5 |
| 1. It is difficult to feel close to others | 1 | 2 | 3 | 4 | 5 |
| 1. Grief has significantly interfered with your ability to work, socialize or function in everyday life | 1 | 2 | 3 | 4 | 5 |
| 1. The grief that you experience would be considered more intense, severe and/or of longer duration that what is expected in your culture. | 1 | 2 | 3 | 4 | 5 |

**Figure 1**

*Japanese model of bereavement based on the socio-interpersonal model*

2.Social affects: emotional control, social emotions

3. Close relationships: upbringing, gender, social support.

4. Culture and society: stigma, changing role of religion

*Note.* This PGD model for Japanese bereaved has 4 layers: 1) Individual symptoms of grief (heart, mind: emotional and cognitive experiences, and body: somatic reactions) 2) Social affects 3) Close relationships 4) wider culture and society. The first layer of the model includes all the internal affective grief reactions of Japanese bereaved adults reported by the key informants; the second layer is the interactional level between the internal affective grief reactions and the social context. The two outer layers of socio/cultural influence (close relationships; culture and society) may serve to filter and shape the experience and expression of grief emotions in Japanese culture.
